# The Higher Parietal Cortical Thickness in Abstinent Methamphetamine Patients Is Correlated With Functional Connectivity and Age of First Usage

**DOI:** 10.3389/fnhum.2021.705863

**Published:** 2021-08-30

**Authors:** Ru Yang, Lei He, Zhixue Zhang, Wenming Zhou, Jun Liu

**Affiliations:** ^1^Department of Radiology, The Second Xiangya Hospital, Central South University, Changsha, China; ^2^Department of Radiology, The First People’s Hospital of Yueyang, Yueyang, China; ^3^Department of Radiology, The First Hospital of Hunan University of Chinese Medicine, Changsha, China

**Keywords:** methamphetamine, long-term abstinence, cortical thickness, addiction, adolescent

## Abstract

**Aim:**

This study aimed to explore the changes of cortical thickness in abstinent methamphetamine (MA) patients compared with healthy controls.

**Materials and Methods:**

Three-tesla structural and functional magnetic resonance imaging (MRI) was obtained from 38 abstinent methamphetamine-dependent (AMD) patients and 32 demographically equivalent healthy controls. The cortical thickness was assessed using FreeSurfer software. General linear model was used to get brain regions with significant different cortical thickness between groups (*p* < 0.05, Monte Carlo simulation corrected). The mean cortical thickness value and functional connectivity with all other brain regions was extracted from those significant regions. Moreover, correlation coefficients were calculated in the AMD group to assess the relations between the mean cortical thickness, functional connectivity and age when they first took MA and the duration of both MA use and abstinence.

**Results:**

The AMD group showed significant cortical thickness increase in one cluster located in the parietal cortex, including right posterior central gyrus, supramarginal gyrus, and superior parietal lobule. In addition, cortical thickness values of those regions were all significant and negatively correlated with the age when patients first used MA. The cortical thickness of right posterior gyrus were positively correlated with its functional connectivities with left middle frontal gyrus and both left and right medial orbitofrontal gyrus.

**Conclusion:**

The higher cortical thickness in the parietal cortex of the AMD group is in agreement with findings in related studies of increased glucose metabolism and gray matter volume. Importantly, the negative correlation between parietal cortical thickness and age of first MA suggested that adolescent brains are more vulnerable to MA’s neurotoxic effect.

## Introduction

Methamphetamine (MA) is an addictive psychoactive drug that has rapid onset and wreaks havoc on the nervous system. It has been widely abused and has become a global public health problem (Var et al., 2016; Darke et al., 2017). According to the Word Drug Reports in 2017, Amphetamines, including amphetamine and methamphetamine, are the second most abused stimulant group worldwide after cannabis (United Nations Office of Drugs Crime, 2017a). MA has dominated the global amphetamines market, accounting for 72% of the global seizures of amphetamines (United Nations Office of Drugs Crime, 2017b). Moreover, various physical illnesses and psychotic disorders can be caused by methamphetamine abuse (Gonzalez et al., 2004; Woods et al., 2005; Cruickshank and Dyer, 2009; London et al., 2015). Worse of all, patients often relapse when they suffer from stress and come across other high risk environments that may trigger MA relapse even after abstinence or treatment (Volkow et al., 2006; McKetin et al., 2012; Brecht and Herbeck, 2014).

Neuroimaging techniques have become powerful methods to study brain structures, functions, and metabolism in MA users. Comprehensive MA related brain structure and function changes have been found (Chang et al., 2005; Ernst and Chang, 2008; Groman et al., 2012) and some can be restored and improved to a certain extent after treatment or abstinence (Groman et al., 2012; Brooks et al., 2016; Choi et al., 2018). Our previous study investigated the gray matter volume difference between abstinent methamphetamine-dependent (AMD) patients and healthy controls (HC) using the voxel-based morphometry (VBM) method (Zhang et al., 2018). “The increased gray matter volumes in the bilateral cerebellum and decreased volumes in the right calcarine and right cuneus were found and suggested abnormal visual and cognitive functions in the AMD patients” (Zhang et al., 2018). Moreover, “the left cerebellum crus GMV was positively correlated with abstinence duration which signaled the cognitive function recovery along with the abstinence.” VBM is an efficient tool to measure structural differences and is sensitive to subtle gray matter alterations. It is more rapid and provides voxel-wise whole brain results compared to manually segmented brain regions in traditional morphometric approaches (Ashburner and Friston, 2000). Therefore, it has been extensively used in psychiatric disorder studies including substance addiction (Morales et al., 2012; Durazzo et al., 2015; Hartwell et al., 2016). As another important structural analysis method, surf-based cortical thickness measurement allows the “regional distribution and quantification of gray matter cortical loss to be specifically assessed in contrast to gyral or lobar volumetric studies which combine gray and white matter within regional volumes” (Rohrer et al., 2009). Hence, cortical thickness can also assess the brain substrates of neurodegenerative disease and provide complementary information to other imaging techniques about neuroanatomy (Rohrer et al., 2009). Therefore it has been commonly used in psychiatry and neural diseases but has not been applied toward the study of MA addiction or abstinence to our knowledge.

In this study, we investigated abnormality of cortical thickness of methamphetamine abstinence patients and its association with functional connectivity and addiction/abstinence variables to provide potential complementary structural biomarkers of MA addiction or abstinence.

## Materials and Methods

### Subjects and MR Imaging Acquisition

Thirty two healthy subjects and 38 AMD subjects were recruited in this study from April 2016 to July 2017. All AMD subjects were recruited from Pingtang Mandatory Detoxification, Changsha City, Hunan Province. The inclusion and exclusion criteria for all subjects in this study were the same as our previous study (Zhang et al., 2018). AMD subjects were diagnosed using the Diagnostic and Statistical Manual on Mental Disorders (DSM-V) and after that had received a long-term (14–25 months) compulsory abstinence. In addition, for all subjects, smoking status, and alcohol consumption were recorded. For every AMD subject, the age when they first used MA, the months of MA use before their most recent abstinence and months of abstinence were also recorded.

Every subject was scanned in a 3T Siemens Skyra MRI scanner equipped with a 32-channel head coil. T1-weighted images and resting-state functional MR images were collected. The detailed MRI scanning sequences and parameters were also identical to the previous study (Zhang et al., 2018).

The study was approved by the Ethics Committees of the Second Xiangya Hospital of Central South University. Confidentiality of personal information and freedom to withdraw from the study were guaranteed.

### Imaging Data Analyses

All MRI images were visually inspected by two radiologists for lesions, structural abnormalities and artifacts. No subjects were excluded.

Cortical reconstructions of the T1-weighted images were performed using FreeSurfer (version 5.3.0)^1^ on a Linux workstation. The detailed steps have been described by related studies (Collins et al., 2017; Perez et al., 2018). For each subject, the gray and white matter boundary derived from automatic segmentation were visually checked and was then used to identify the pial surface with a deformable surface algorithm. Cortical thickness was measured as the distance between the white matter and pial surfaces. After construction, images were then morphed and registered to an average spherical space where gyral and sulcal features were optimally aligned. Individual measures were then transformed into the average space. Cortical thickness maps were then smoothed with a 15 mm half-maximum full-width Gaussian kernel.

Functional images processing was performed with DPABI (a toolbox for Data Processing and Analysis of Brain Imaging). After preprocessing including slice timing, realign, normalization and nuisance covariates regression, functional connectivity was calculated on Anatomic-Automatic-Labeling (AAL) template.

### Statistical Analysis

Demographics were compared between AMD and healthy control groups with SPSS 21.0. Age and years of education were compared using two-sample *t*-test while smoking status and alcohol consumption were tested using Fisher exact test. The significance level was set to *p* < 0.05.

QDEC tool in FreeSurfer was utilized to compare cortical thickness between two groups using a 2-class general linear model (GLM). Multiple comparisons were corrected using Monte Carlo simulation method with an initial vertex-wise threshold of *p* < 0.01 and vertex level corrected to *p* < 0.05.

Mean values were then extracted from brain regions which showed significantly different cortical thickness between the two groups. In the AMD group, we calculated the correlation coefficients of those mean cortical thickness values with patients’ age when they first used MA, the total months of MA use and abstinence. The significance level was set to *p* < 0.05.

The functional connectivity of significant region to any other regions on AAL template was also extracted. The correlation coefficients between these connectivity values with cortical thickness values were calculated. The significance level was set to *p* < 0.05.

## Results

### Demographics

Our study included 38 AMD patients and 32 healthy subjects. As showed in Table 1, there were no significant differences between the two groups in age, years of education, smoking status or alcohol consumption.

**TABLE 1:** Demographic information and characterization.

Group	AMD	HC	*p*
N	38	32	
Age/year	33.1 ± 6.0	34.5 ± 7.0	0.353^a^
Education/year	8.7 ± 2.1	9.6 ± 2.4	0.113^a^
Smoking (Yes/No)	37/1	30/2	0.589^b^
Drinking (Yes/No)	13/25	8/24	0.443^b^
Age of first MA use	26.0 ± 6.9		
Months of MA use	64.2 ± 34.2		
Months of abstinence	19.1 ± 2.7		

*^*a*^Two-sample t-test.*

*^*b*^Fisher exact test. Significant level was set at p < 0.05. There are no statistically significant differences between AMD and HC group based on demographic information and characterization.*

### Cortical Thickness Analysis Results

**FIGURE 1:**
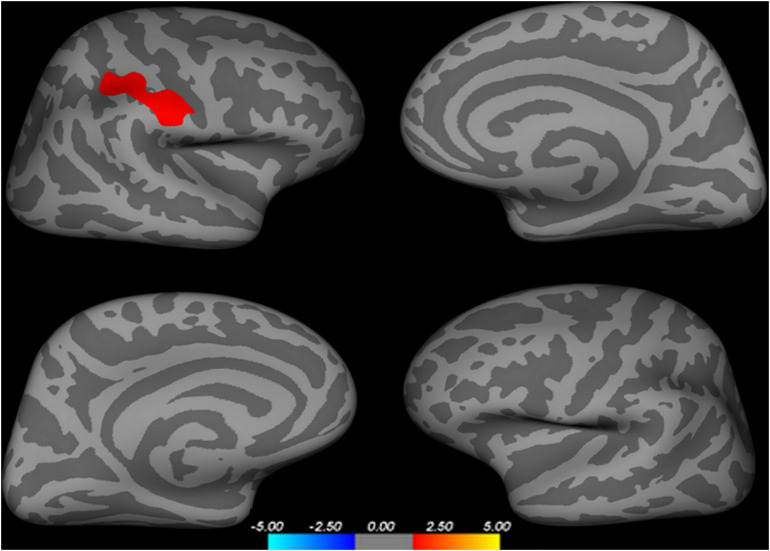
Regions with increased cortex thickness in AMD group.

In comparison with the HC group, the AMD group showed significant cortical thickness increase in one cluster in parietal cortex. The detailed location of the cluster was defined by overlapping it with AAL template. It contains three parts including right posterior central gyrus, supramarginal gyrus, and superior parietal lobule (Table 2 and Figure 1).

**TABLE 2:** Regions with increased cortical thickness in AMD group compared with HC group.

Brain region (AAL)	Volume (mm^3^)	*p*	Peak talairach coordinates		
			
			X	Y	Z
Right posterior central gyrus (peak location)	1347.13	0.0474*	54.2	–14.6	34.4

*Statistical threshold was p < 0.05 corrected for multiple comparisons by Monte Carlo simulation method; Coordinates are located in Talairach space. AAL, Anatomic-Automatic-Labeling template.* *p < 0.05.

### Correlation Analyses

**FIGURE 2:**
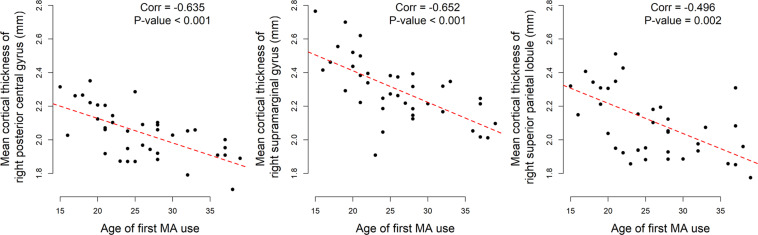
Significant correlations between age of first MA use and mean cortical thickness of abnormal regions in AMD group.

In the AMD group, the mean cortical thickness of all three regions were significantly negatively correlated with the age of first MA usage: right posterior central gyrus, *r* = –0.635, *p* < 0.001; right supramarginal gyrus, *r* = –0.652, *p* < 0.001; right superior parietal lobule, *r* = –0.496, *p* = 0.002. No significant correlations were found between cortical thickness and months of MA use or months of abstinence (Figure 2).

Intuitively, the association between cortical thickness of abnormal regions in AMD patients and the age of first MA use could be affected by the duration of MA use. Therefore, partial correlation coefficients were calculated to exclude the effect of MA use duration. Those three mean cortical thicknesses were still strongly correlated to the age of first MA use: Right posterior central gyrus, *r* = –0.617, *p* < 0.001; right supramarginal gyrus, *r* = –0.644, *p* < 0.001; right superior parietal lobule, *r* = –0.588, *p* < 0.001.

Moreover, the cortical thickness of right posterior gyrus were positively correlated with its functional connectivities to three regions including left middle frontal gyrus (*r* = 0.324, *p* = 0.047), right medial orbitofrontal gyrus (*r* = 0.397, *p* = 0.014) and left medial orbitofrontal gyrus (*r* = 0.334, *p* = 0.041).

## Discussion

In this study, we adopted the surface-based cortical thickness method to investigate the abnormal brain structure in abstinent methamphetamine-dependent patients. Increased cortical thickness was found in one cluster located in the right parietal cortex, including right posterior central gyrus, supramarginal gyrus, and superior parietal lobule. In addition, mean cortical thickness values of those regions were all strongly negatively correlated with age of first MA use in AMD patients. Moreover, the cortical thickness of right posterior gyrus were positively correlated with its functional connectivities left middle frontal gyrus, right medial orbitofrontal gyrus, and left medial orbitofrontal gyrus.

The higher cortical thickness in parietal cortex of the AMD group agreed with previous studies that observed increased glucose metabolism in both short and long term abstinent MA patients (Volkow et al., 2001; Berman et al., 2008) and increased gray matter (Jernigan et al., 2005) in abstinence methamphetamine patients, and the increased glucose metabolism in high dose MA treatment rats’ brains without abstinence (Thanos et al., 2016). The parietal cortex was found to be especially sensitive to methamphetamine neurotoxicity (Volkow et al., 2001). The increased cortical thickness found in this study could be explained by the growing numbers of microglia and astrocytes, which could driven by MA abuse (LaVoie et al., 2004) and were thought to increase the cerebral glucose metabolic. Moreover, the activated microglia were linked to vasculature outside of neurodegeneration regions (Bowyer et al., 2017), which could also increase the cortical thickness in parietal cortex. Although increased microglial activation along with increased brain volume were found after chronic MA treatment (Thanos et al., 2016), their causal relations have not yet been proven.

MA users have varied decision-making changes and the parietal cortex has been shown to be critical for it (Bowyer et al., 2007). Specifically, the parietal cortex activation levels have been found to be correlated to decision making of uncertainty in MA patients (Paulus et al., 2003). The parietal cortex metabolism in MA users was correlated with Grooved pegboard tasks performance, which is also involved with decision-making (Volkow et al., 2001). In addition, gene expression changes related to synaptic plasticity were also found in the parietal cortex and may be related to these behavioral outcomes (Paulus et al., 2001). Moreover, the middle frontal gyrus and medial orbitofrontal cortex are both important areas for decision making. That their connections with right posterior gyrus were positively correlated with its cortical thickness also implied the parietal cortex plays an essential role in MA addiction.

The peak intensity value of the significant cluster in our study was located in the right posterior central gyrus, which contains the primary somatosensory cortex. Besides dopaminergic and serotonergic terminals, a study on adult rats indicated that MA also has the neurotoxic effect on glutamatergic neurons in the somatosensory cortex (Pu et al., 1996). Reactive microgliosis was also observed in the somatosensory cortex (LaVoie et al., 2004).

Importantly, we found that cortical thickness of significant regions located in the parietal cortex were negatively correlated with age of first MA use with or without excluding the effect of MA use duration. In other words, the younger the patient was when starting to abuse MA, the thicker those regions were than in healthy patients no matter how long they used MA. A study by Jernigan et al. (2005) showed that nucleus accumbens volume increase associated with MA dependence has a larger effect on younger MA patients. Also, it was reported that the age when MA was first used was positively related with intracranial volume (Huckans et al., 2010). “Adolescence is a critical period of brain development as the brain undergoes dynamic synaptic reorganization and myelination” (Castellanos et al., 1999). On the one hand, environmental insults can affect brain development and cause irreversible damage to the adolescent brain (Castellanos et al., 1999; Rapoport and Brain, 2008). On the other hand, the adolescent brain can recover more effectively from lesions for its greater neuroplasticity (Lyoo et al., 2015). In the parietal cortex, increased glucose metabolism was found in brains with and without abstinence after MA treatment, and the increased gray matter volume was found in both short and long term MA abstinent patients. We speculated that the abnormal parietal cortex was mainly caused by MA exposure before abstinence. The negative correlation between age of first MA use and the cortical thickness could be interpreted as: adolescent brains are more vulnerable to MA neurotoxic effects that cause irreversible damage even after a long-term abstinence.

## Limitation

In this study, we found higher cortical thickness in parietal cortex of the AMD group, which is agreed with the increased glucose metabolism and gray matter in related studies. However, the underlying mechanisms are still not clear. Future studies are encouraged to explore the causal relation between increased microglial activation and brain volume change or MA usage. Moreover, that the negative correlation between age of first MA use and the cortical thickness also need to be validated and explained by researches from other modalities.

## Data Availability Statement

The raw data supporting the conclusions of this article will be made available by the authors, without undue reservation.

## Ethics Statement

The studies involving human participants were reviewed and approved by the Ethics Committee of the Second Xiangya Hospital, Central South University. The patients/participants provided their written informed consent to participate in this study.

## Author Contributions

RY, WZ, and JL conceptualized and designed the research. ZZ collected the demographics and MRI data. LH analyzed MRI data and undertook the statistical analysis with RY. RY and LH wrote the first draft. RY contributed to final manuscript including editing figures, tables, and format. All authors critically reviewed the content and approved the final version for publication.

## Conflict of Interest

The authors declare that the research was conducted in the absence of any commercial or financial relationships that could be construed as a potential conflict of interest.

## Publisher’s Note

All claims expressed in this article are solely those of the authors and do not necessarily represent those of their affiliated organizations, or those of the publisher, the editors and the reviewers. Any product that may be evaluated in this article, or claim that may be made by its manufacturer, is not guaranteed or endorsed by the publisher.

## Footnotes

1
https://surfer.nmr.mgh.harvard.edu/
